# Supplementary material for: FLInt: single shot safe harbor transgene integration via Fluorescent Landmark Interference
Source: G3 (Bethesda). 2023 Feb 20;13(5):jkad041. doi: 10.1093/g3journal/jkad041 (PMC10151404; doi:10.1093/g3journal/jkad041)
Supplement: jkad041_Supplementary_Data [file jkad041_supplementary_data.zip › Supplemental_Data_1_G3-2022-404006.pdf]

Template Alignment: C55B7.3 sequence alignment

AAGAAAGTGTTTCGAATACTTCCCGAGCAAGAAGGGTGATGTGATGGACTTTGAGGAAGGTGGTCAGAAGATCTCAGTTAAATGCGAATCTTCGGTGACG

off-target site

template sequence C55B7.3 sequence

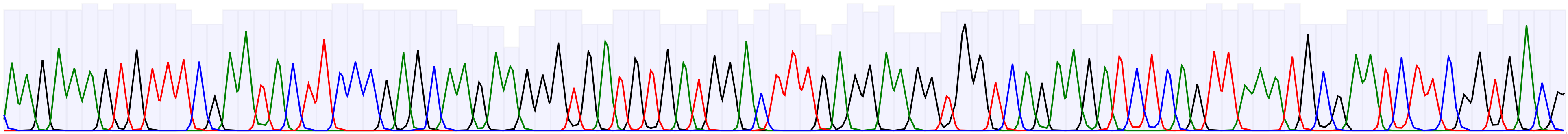

AAGAAAGTGTTTCGAATACTTCCCGAGCAAGAAGGGTGATGTGATGGACTTTGAGGAAGGTGGTCAGAAGATCTCAGTTAAATGCGAATCTTCGGTGACG

aligned sequence mirIs123 [*oxTi553] V

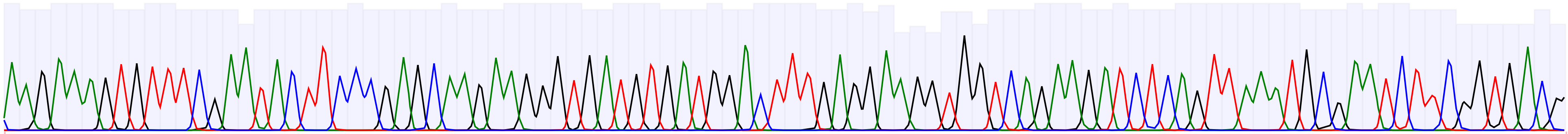

AAGAAAGTGTTTCGAATACTTCCCGAGCAAGAAGGGTGATGTGATGGACTTTGAGGAAGGTGGTCAGAAGATCTCAGTTAAATGCGAATCTTCGGTGACG

aligned sequence mirIs124 [*oxTi553] V

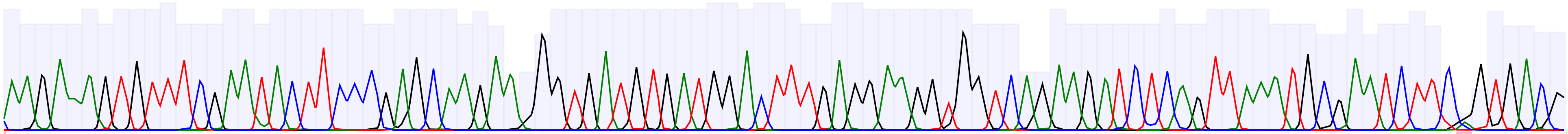

AAGAAAGTGTTTCGAATACTTCCCGAGCAAGAAGGGTGATGTGATGGACTTTGAGGAAGGTGGTCAGAAGATCTCAGTTAAATGCGAATCTTCAGTGACG

aligned sequence mirIs125 [*oxTi553] V

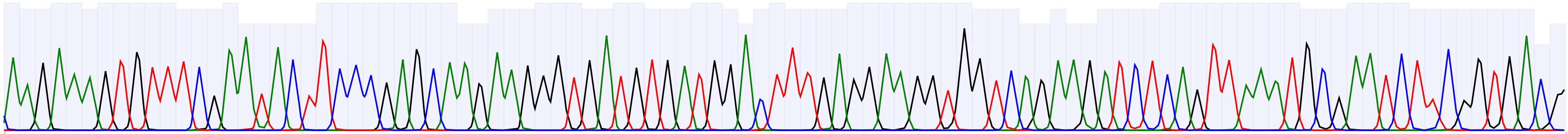

AAGAAAGTGTTTCGAATACTTCCCGAGCAAGAAGGGTGATGTGATGGACTTTGAGGAAGGTGGTCAGAAGATCTCAGTTAAATGCGAATCTTCGGTGACG

aligned sequence mirIs126 [*oxTi553] V

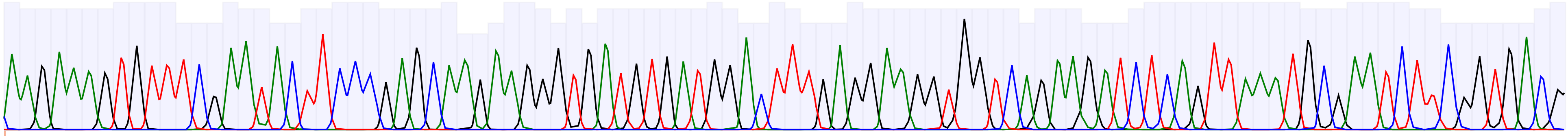

AAGAAAGTGTTTCGAATACTTCCCGAGCAAGAAGGGTGATGTGATGGACTTTGAGGAAGGTGGTCAGAAGATCTCAGTTAAATGCGAATCTTCGGTGACG

aligned sequence mirIs127 [*oxTi677] II

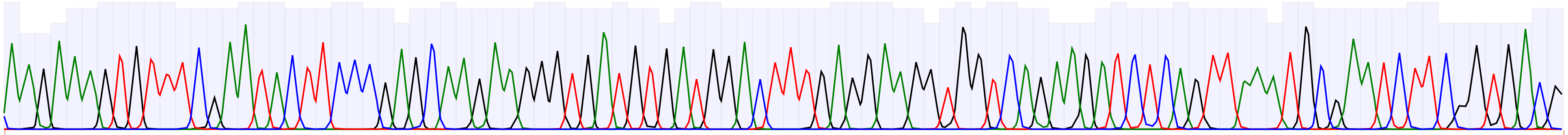

AAGAAAGTGTTTCGAATACTTCCCGAGCAAGAAGGGTGATGTGATGGACTTTGAGGAAGGTGGTCAGAAGATCTCAGTTAAATGCGAATCTTCGGTGACG

aligned sequence mirIs128 [*oxTi677] II

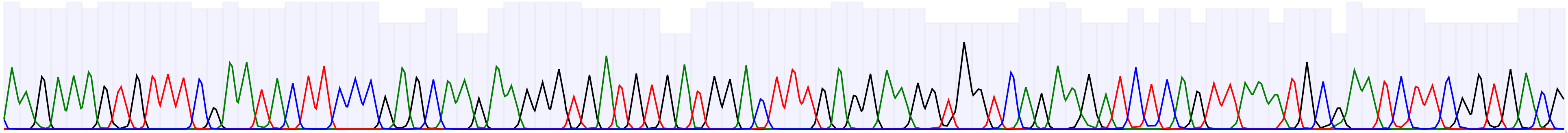

AAGAAAGTGTTTCGAATACTTCCCGAGCAAGAAGGGTGATGTGATGGACTTTGAGGAAGGTGGTCAGAAGATCTCAGTTAAATGCGAATCTTCGGTGACG

aligned sequence mirIs129 [*oxTi677] II

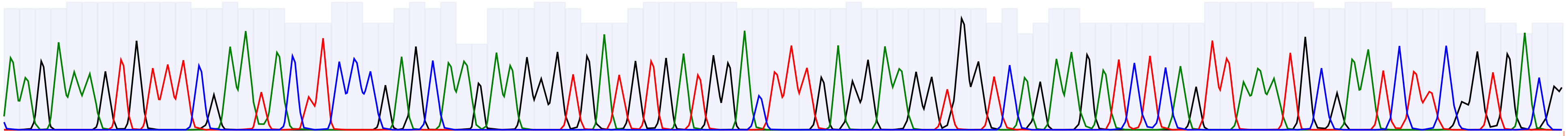

AAGAAAGTGTTTCGAATACTTCCCGAGCAAGAAGGGTGATGTGATGGACTTTGAGGAAGGTGGTCAGAAGATCTCAGTTAAATGCGAATCTTCGGTGACG

aligned sequence mirIs130 [*oxTi546] III

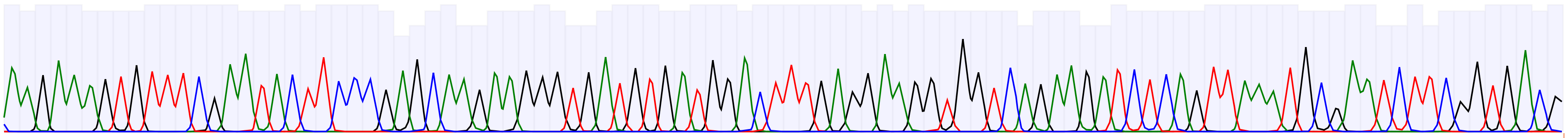

AAGAAAGTGTTTCGAATACTTCCCGAGCAAGAAGGGTGATGTGATGGACTTTGAGGAAGGTGGTCAGAAGATCTCAGTTAAATGCGAATCTTCGGTGACG

aligned sequence mirIs131 [*oxTi546] III

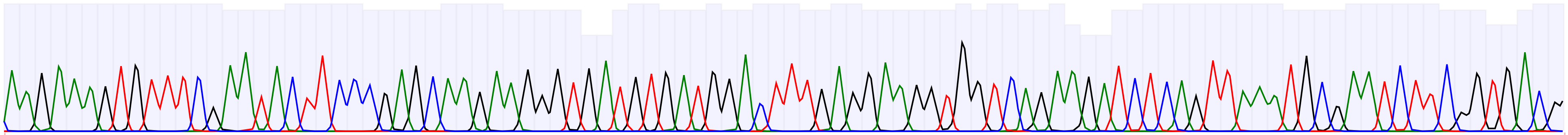

AAGAAAGTGTTTCGAATACTTCCCGAGCAAGAAGGGTGATGTGATGGACTTTGAGGAAGGTGGTCAGAAGATCTCAGTTAAATGCGAATCTTCGGTGACG

aligned sequence mirIs132 [*oxTi546] III

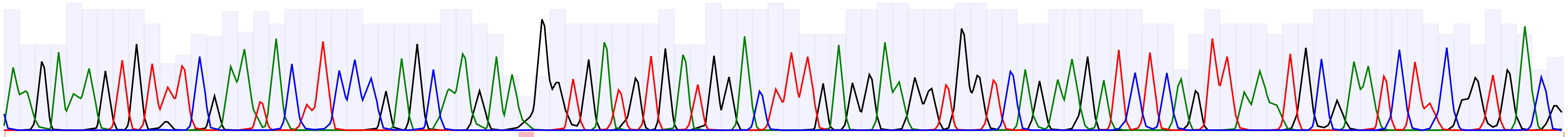

AAGAAAGTGTTTCGAATACTTCCCGAGCAAGAAAGGTGATGTGATGGACTTTGAGGAAGGTGGTCAGAAGATCTCAGTTAAATGCGAATCTTCGGTGACG

aligned sequence mirIs133 [*oxTi546] III

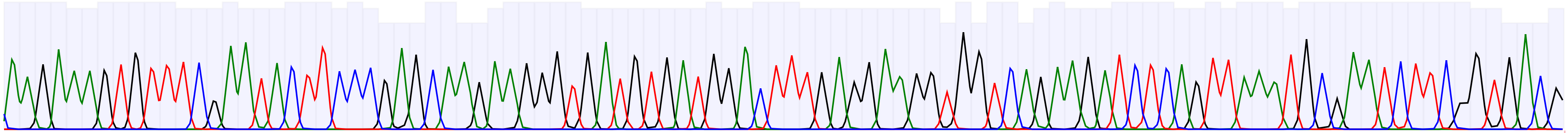

AAGAAAGTGTTTCGAATACTTCCCGAGCAAGAAGGGTGATGTGATGGACTTTGAGGAAGGTGGTCAGAAGATCTCAGTTAAATGCGAATCTTCGGTGACG

aligned sequence mirIs134 [*oxTi553] V

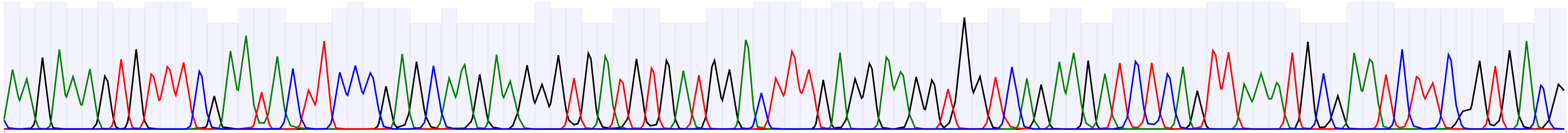

AAGAAAGTGTTTCGAATACTTCCCGAGCAAGAAGGGTGATGTGATGGACTTTGAGGAAGGTGGTCAGAAGATCTCAGTTAAATGCGAATCTTCGGTGACG

aligned sequence mirIs135 [*oxTi553] V

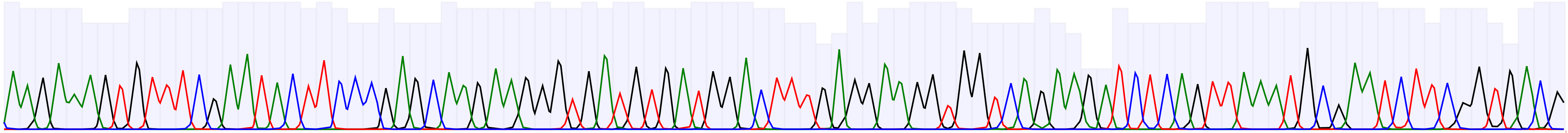

AAGAAAGTGTTTCGAATACTTCCCGAGCAAGAAGGGTGATGTGATGGACTTTGAGGAAGGTGGTCAGAAGATCTCAGTTAAATGCGAATCTTCGGTGACG

aligned sequence mirIs136 [*oxTi553] V

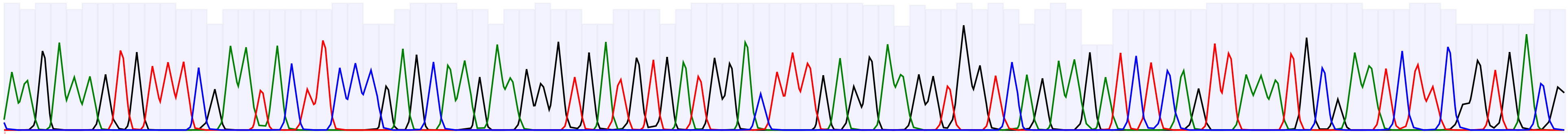

AAGAAAGTGTTTCGAATACTTCCCGAGCAAGAAGGGTGATGTGATGGACTTTGAGGAAGGTGGTCAGAAGATCTCAGTTAAATGCGAATCTTCGGTGACG

aligned sequence mirIs137 [*oxTi553] V
