# Supplementary material for: FLInt: single shot safe harbor transgene integration via Fluorescent Landmark Interference
Source: G3 (Bethesda). 2023 Feb 20;13(5):jkad041. doi: 10.1093/g3journal/jkad041 (PMC10151404; doi:10.1093/g3journal/jkad041)
Supplement: jkad041_Supplementary_Data [file jkad041_supplementary_data.zip › Supplemental_Material_and_Figures_G3-2022-404006.pdf]

# Supplemental Materials

# Contents

**1 Supplemental Figures** **2**

1.1 Figure S1 . . . . . . . . . . . . . . . . . . . . . . . . . . . . . . . . . . . . . 2

1.2 Figure S2 . . . . . . . . . . . . . . . . . . . . . . . . . . . . . . . . . . . . . 3

1.3 Figure S3 . . . . . . . . . . . . . . . . . . . . . . . . . . . . . . . . . . . . . 4

1.4 Figure S4 . . . . . . . . . . . . . . . . . . . . . . . . . . . . . . . . . . . . . 5

**2 Supplemental Tables** **6**

2.1 Table S1 . . . . . . . . . . . . . . . . . . . . . . . . . . . . . . . . . . . . . . 6

2.2 Table S2 . . . . . . . . . . . . . . . . . . . . . . . . . . . . . . . . . . . . . . 6

2.3 Table S3 . . . . . . . . . . . . . . . . . . . . . . . . . . . . . . . . . . . . . . 6

2.4 Table S4 . . . . . . . . . . . . . . . . . . . . . . . . . . . . . . . . . . . . . . 6

2.5 Table S5 . . . . . . . . . . . . . . . . . . . . . . . . . . . . . . . . . . . . . . 6

2.6 Table S6 . . . . . . . . . . . . . . . . . . . . . . . . . . . . . . . . . . . . . . 6

2.7 Table S7 . . . . . . . . . . . . . . . . . . . . . . . . . . . . . . . . . . . . . . 6

**3 Supplemental Data File 1** **7**

# 1 Supplemental Figures

## 1.1 Figure S1

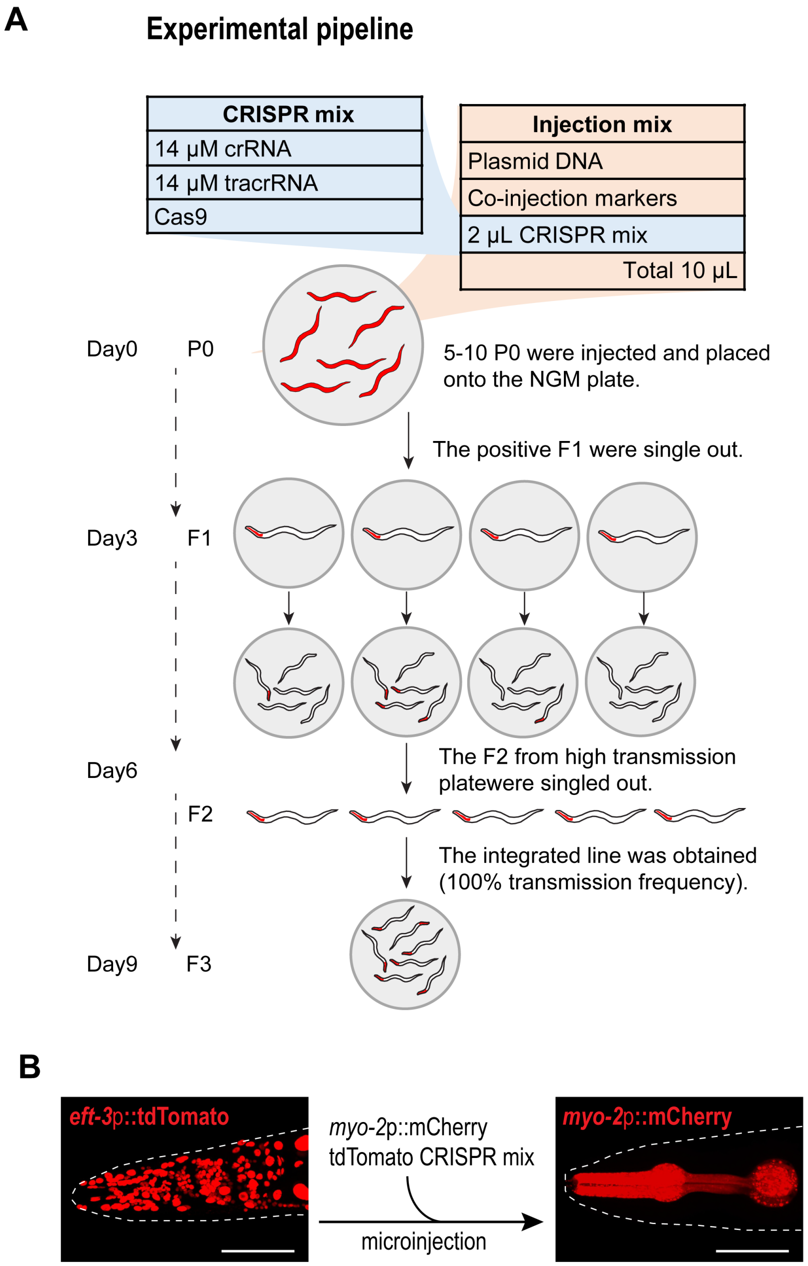


**Supp. Fig. 1.** Experimental pipeline for FLInt integrations
**A:** General procedure for transgene integration into a tdTomato locus. **B:** Representative photograph of an animal before and after successful integration. The correlation of a loss in red nuclear signal together with transgene fluorescence indicates successful integration.

## 1.2 Figure S2

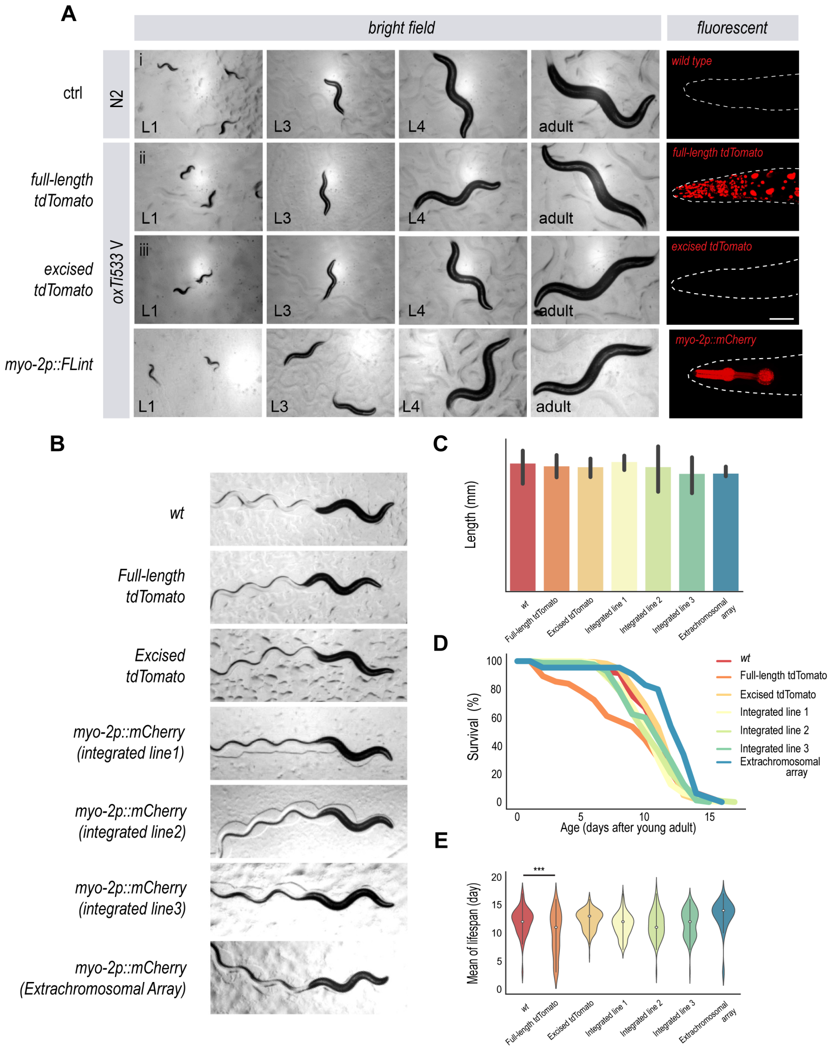


**Supp. Fig. 2.** Health and lifespan of FLInt integrants
**A:** Developmental time course of (i) wt animals, (ii) original strain with landing site (iii) recombined animals with no integration and (iv) FLInt animals from L1-adult day 1 and their fluorescent signal. **B:** Tracks of individual genotypes used to demonstrate the FLInt strategy. **C:** Body length of the employed genotypes. N=3 independent replicates. All conditions are p>0.05, as tested with Anova, Tukey-corrected for multiple comparisons. **D:** Lifespan curve of the FLInt animals. **E:** Lifespan distribution of the FLInt animals. p-values derived from a two sided Anova, Tukey-corrected test for multiple comparisons.

## 1.3 Figure S3

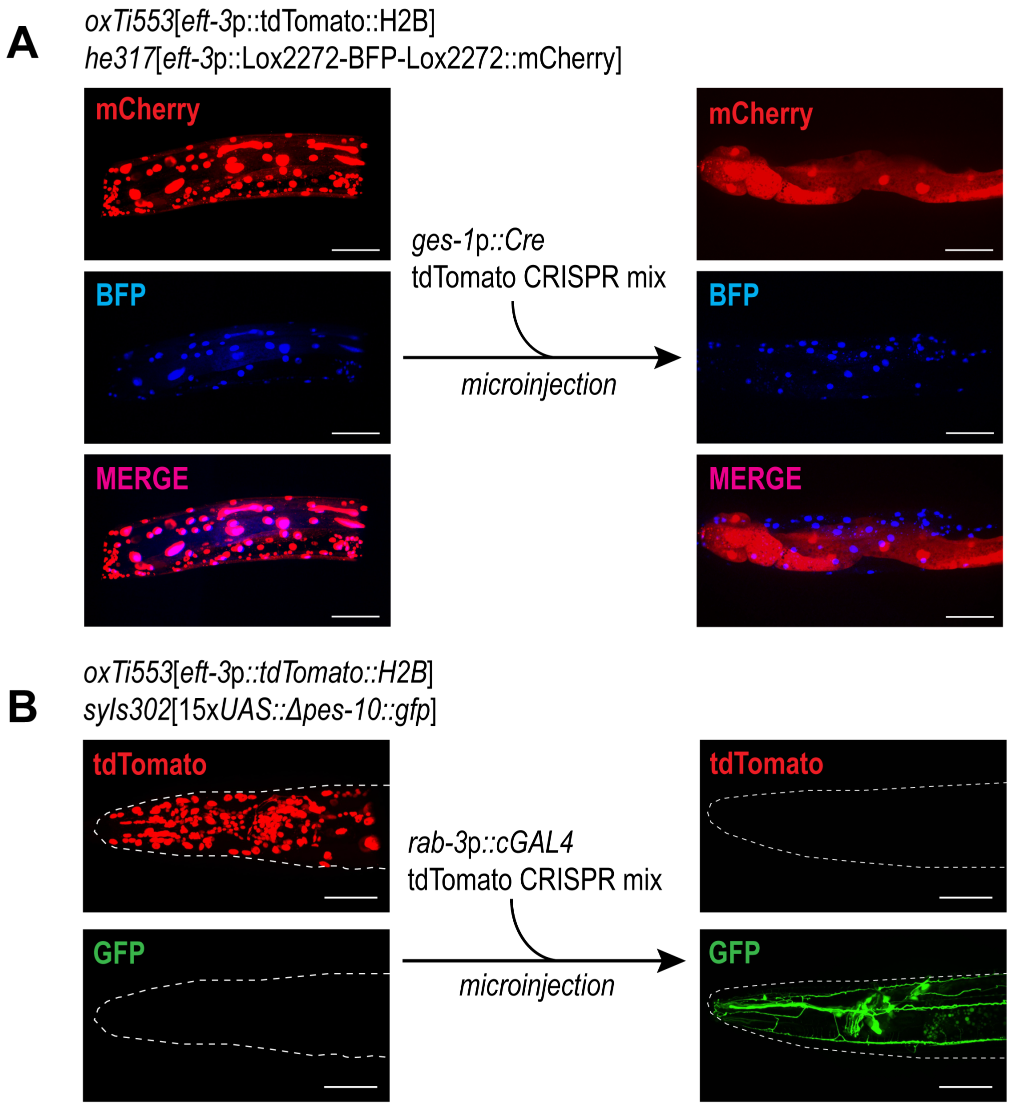


**Supp. Fig. 3.** Using tdTomato FLInt as a co-transformation marker in drive/effector binary systems
**A:** Integration of a recombinase enzyme (*ges-1*p::CRE) without need of a co-injection marker in *loxP* recombination marker background and screening by the BFP-to-mCherry color switch in CRE-expressing tissue (intestine). **B:** Integration of a transcription factor (*rab-3*p::cGAL4) in *UAS::gfp* background strain screening the GFP expression in *C. elegans* nervous system to isolate positive events.

## 1.4 Figure S4

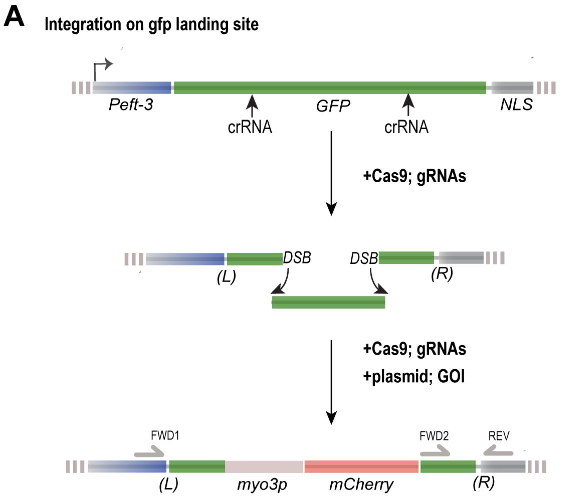


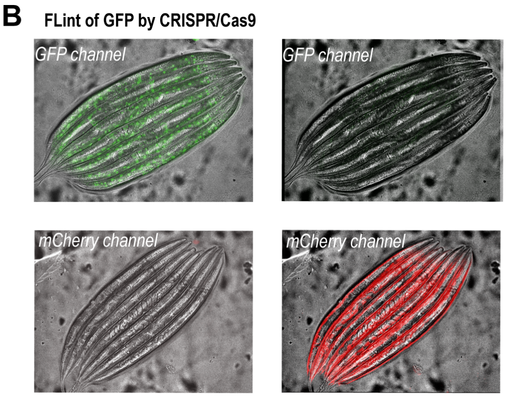


| | red | non-red |
|---|---|---|
| green | 34+/-16 | 9+/-11 |
| non-green | 8+/-7 | 48+/-30 |

C

| Tukey's Multiple Comparison Test | Mean Diff. | q | Significant? P < 0.05? |
|---|---|---|---|
| red+green vs green | 26.31 | 2.816 | No |
| red+green vs red | 27.87 | 2.982 | No |
| red+green vs none | -12.2 | 1.306 | No |
| green vs red | 1.555 | 0.1664 | No |
| green vs none | -38.52 | 4.122 | No |
| red vs none | -40.07 | 4.288 | Yes |

**Supp. Fig. 4.** FLInt of GFP as a target site
**A:** Schematic of the single copy loci and the gene replacement strategy. **B:** Representative outcome of the experiment is visualized by loss of GFP fluorescence and appearance of the transgene expression (BWM::mCherry). Below, table with number of observations (out of 100 animals; mean ± standard deviation) in tdTomato and GFP crRNA injected animals. **C:** Table with the Tukey-corrected ANOVA results for multiple comparisons of the outcome.

# 2 Supplemental Tables

## 2.1 Table S1

Detailed summary of the integration efficiency of all FLint loci tested with a standardized injection experiment in this study.

## 2.2 Table S2

Summary and characteristics of strains generated using FLInt and other strains used in this study.

## 2.3 Table S3

Plasmid(s) used and generated in this study.

## 2.4 Table S4

Sequences of the DNA primer(s)

## 2.5 Table S5

All crRNA(s) used in this study.

## 2.6 Table S6

Homology directed repair templates.

## 2.7 Table S7

tdTomato CRISPR mix preparation

# 3 Supplemental Data File 1

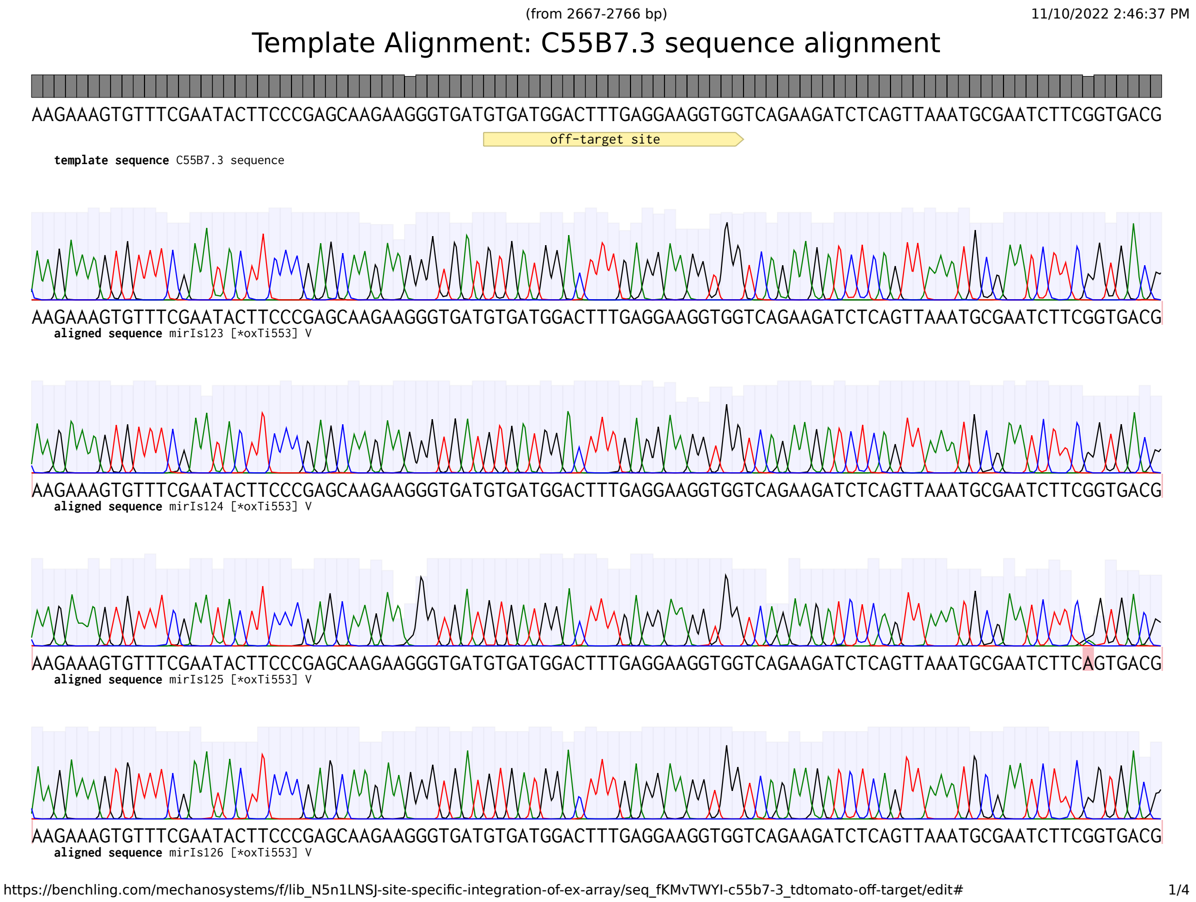


**Supp. Data 1.** Sequencing results for predicted off-target site in C55B7.3
The employed tdTomato crRNAs are predicted to recognize the C55B7.3 sequence with four mismatched bases. However, no sequence defects have been detected in a total of 50 edited strains, 9 of which are shown here.
