# Supplementary material for: FLInt: single shot safe harbor transgene integration via Fluorescent Landmark Interference
Source: G3 (Bethesda). 2023 Feb 20;13(5):jkad041. doi: 10.1093/g3journal/jkad041 (PMC10151404; doi:10.1093/g3journal/jkad041)
Supplement: jkad041_Supplementary_Data [file jkad041_supplementary_data.zip › Supplemental_Table_S1_G3-2022-404006.pdf]

Chr.	tdTomato insertion locus	Parent strains	Experiment	No. of injected P0	No. of positive F1	No. of high transmission F2	No. of integrated line	Integration frequency*	average
LG1	I:22.3	EG7846	1	20	37	5	0	0%	**2%**
LG1	I:22.3	EG7846	2	20	16	7	0	0%	
LG1	I:22.3	EG7846	3	50	17	2	1	6%	
LG1	I:22.3	EG7846	4	20	52	8	0	0%	
LG1	I:22.3	EG7846	5	50	80	27	5	6%	
LG1	I:22.3	EG7846	6	30	82	8	2	2%	
LG1	I:22.3	EG7846	7	50	26	2	0	0%	
LG1	I:1.23	EG7835	8	10	8	1	1	13%	**6%**
LG1	I:1.23	EG7835	9	10	60	11	2	3%	
LG1	I:1.23	EG7835	10	10	55	8	2	4%	
LG2	II:-12.71	EG7860	11	20	20	2	1	5%	**4.73%**
LG2	II:-12.71	EG7860	12	20	11	2	1	9%	
LG2	II:-12.71	EG7860	13	20	59	8	1	2%	
LG2	II:-12.71	EG7860	14	40	32	4	1	3%	
LG2	II:-0.38	EG7866	15	10	38	8	1	3%	**6%**
LG2	II:-0.38	EG7866	16	10	38	5	4	11%	
LG2	II:-0.38	EG7866	17	10	16	4	1	6%	
LG3	III:11.8	EG7900	18	20	25	3	1	4%	**5%**
LG3	III:11.8	EG7900	19	20	15	7	1	7%	
LG3	III:11.8	EG7900	20	20	46	7	2	4%	
LG3	III:11.8	EG7900	21	30	51	6	2	4%	
LG3	III:1.23	EG7898	22	10	35	6	2	6%	**6%**
LG3	III:1.23	EG7898	23	10	84	17	4	5%	
LG3	III:1.23	EG7898	24	10	57	7	4	7%	
LG4	IV:-26.93	EG7905	25	20	18	7	0	0%	**0%**
LG4	IV:-26.93	EG7905	26	20	15	2	0	0%	
LG4	IV:-26.93	EG7905	27	20	45	9	0	0%	
LG4	IV:-26.93	EG7905	28	50	55	9	0	0%	
LG4	IV:-26.93	EG7905	29	40	65	10	0	0%	
LG4	IV:-26.93	EG7905	30	30	78	7	0	0%	
LG4	IV:0.09	EG7911	31	10	20	1	0	0%	**2%**
LG4	IV:0.09	EG7911	32	10	43	7	2	5%	

LG4	IV:0.09	EG7911	33	10	30	3	0	0%	
LG5	V:0.29	EG7944	34	20	26	8	1	4%	**5%**
LG5	V:0.29	EG7944	35	20	9	3	2	22%	
LG5	V:0.29	EG7944	36	20	32	2	1	3%	
LG5	V:0.29	EG7944	37	50	25	5	1	4%	
LG5	V:0.29	EG7944	38	52	40	11	2	5%	
LG5	V:0.29	EG7944	39	45	69	30	2	3%	
LG5	V:0.29	EG7944	40	50	18	3	0	0%	
LG5	V:0.29	EG7944	41	60	38	6	1	3%	
LG5	V:0.29	EG7944	42	50	40	8	2	5%	
LGX	X:-4.88	EG7985	43	20	23	6	0	0%	**1%**
LGX	X:-4.88	EG7985	44	20	27	8	0	0%	
LGX	X:-4.88	EG7985	45	20	27	5	0	0%	
LGX	X:-4.88	EG7985	46	50	15	0	0	0%	
LGX	X:-4.88	EG7985	47	40	46	6	2	4%	
LGX	X:-4.88	EG7985	48	20	27	5	0	0%	
LGX	X:0.19	EG7989	49	10	49	9	0	0%	**6%**
LGX	X:0.19	EG7989	50	10	60	8	7	12%	
LGX	X:0.19	EG7989	51	10	44	5	3	7%	
