# Supplementary material for: FLInt: single shot safe harbor transgene integration via Fluorescent Landmark Interference
Source: G3 (Bethesda). 2023 Feb 20;13(5):jkad041. doi: 10.1093/g3journal/jkad041 (PMC10151404; doi:10.1093/g3journal/jkad041)
Supplement: jkad041_Supplementary_Data [file jkad041_supplementary_data.zip › Supplemental_Table_S2_G3-2022-404006.pdf]

Strain	Genotype	Allele	crRNA	injected DNA	total DNA
**MSB848**	*unc-119(ed3) III; mirIs113 [*oxTi553] V*	*unc-119(ed3); mirIs113[unc-122p::GFP; myo-2p::mCherry *oxTi553 [eft-3p::tdTomato::H2B::unc-54 3'UTR + Cbr-unc-119(+)]] V*	14 ng/µL crRNA(tdTomato)	50 ng/µL pCFJ68 + 2 ng/µL pMK73	52 ng/µL
**MSB864**	*unc-119(ed3) III; mir114 [*oxTi553] V*	*unc-119(ed3) III; mirIs114 [unc-122p::GFP; myo-2p::mCherry *oxTi553 [eft-3p::tdTomato::H2B::unc-54 3'UTR + Cbr-unc-119(+)] V*	14 ng/µL crRNA(tdTomato)	50 ng/µL pCFJ68 + 2 ng/µL pMK73	52 ng/µL
**MSB884**	*unc-119(ed3) III; mirIs78 [*oxTi553] V*	*unc-119(ed3) III; mirIs78 [cct-2p::mtagBFP::SPE-15::spectrin::cryo2lig::wrmScarlet(1-10) + mec-4p::TRP-4::wrmScarlet(11) + mec-4p::RGECO1a + myo-2p::mCherry+ 1kb DNA ladder *oxTi553 [eft-3p::tdTomato::H2B::unc-54 3'UTR + Cbr-unc-119(+)]] V*	2.8 ng/µL crRNA(tdTomato)	10 ng/µL pNM10 + 10 ng/µL pNM11 + 10 ng/µL pNM12 + 2 ng/µL pMK73 + 68 ng/µL DNA ladder	100 ng/µL
**MSB885**	*unc-119(ed3) III; mirIs79 [*oxTi553] V*	*unc-119(ed3) III; mirIs79 [cct-2p::mtagBFP::SPE-15::spectrin::cryo2lig::wrmScarlet(1-10) + mec-4p::TRP-4::wrmScarlet(11) + mec-4p::RGECO1a + myo-2p::mCherry + 1kb DNA ladder *oxTi553 [eft-3p::tdTomato::H2B::unc-54 3'UTR + Cbr-unc-119(+)]] V*	2.8 ng/µL crRNA(tdTomato)	10 ng/µL pNM10 + 10 ng/µL pNM11 + 10 ng/µL pNM12 + 2 ng/µL pMK73 + 68 ng/µL DNA ladder	100 ng/µL
**MSB886**	*unc-119(ed3) III; mirIs80 [*oxTi553] V*	*unc-119(ed3) III; mirIs80 [ges-1p::Cre + myo-2p::mCherry + 1kb DNA ladder *oxTi553 [eft-3p::tdTomato::H2B::unc-54 3'UTR + Cbr-unc-119(+)]] V*	2.8 ng/µL crRNA(tdTomato)	20 ng/µL pNM13 + 2 ng/µL pMK73 + 78 ng/µL DNA ladder	100 ng/µL
**MSB896**	*mirIs81 [*oxTi553] V; ljSi123; lite-1(ce314)*	*mirIs81 [mec-4p::hygromycin resistance + myo-2p::mCherry + 1kb DNA ladder *oxTi553 [eft-3p::tdTomato::H2B::unc-54 3'UTR + Cbr-unc-119(+)]] V; ljSi123[mec-7p:GCaMP6s::SL2::tagRFP] II; lite-1(ce314)*	2.8 ng/µL crRNA(tdTomato)	20 ng/µL pSDV2 + 2 ng/µL pMK73 + 78 ng/µL DNA ladder	100 ng/µL
**MSB897**	*mirIs82 [*oxTi553] V; ljSi123; lite-1(ce314)*	*mirIs82 [mec-4p::hygromycin resistance + myo-2p::mCherry + 1kb DNA ladder *oxTi553 [eft-3p::tdTomato::H2B::unc-54 3'UTR + Cbr-unc-119(+)]] V; ljSi123[mec-7p:GCaMP6s::SL2::tagRFP] II; lite-1(ce314)*	2.8 ng/µL crRNA(tdTomato)	20 ng/µL pSDV2 + 2 ng/µL pMK73 + 78 ng/µL DNA ladder	100 ng/µL
**MSB898**	*unc-119(ed3) III; mirIs83 [*oxTi553] V*	*unc-119(ed3) III; mirIs83[cct-2p::mtagBFP::SPE-15::spectrin::cryo2lig::wrmScarlet(1-10) + mec-4p::TRP-4::wrmScarlet(11) + mec-4p::RGECO1a + myo-2p::mCherry+ 1kb DNA ladder *oxTi553 [eft-3p::tdTomato::H2B::unc-54 3'UTR + Cbr-unc-119(+)]] V*	2.8 ng/µL crRNA(tdTomato)	20 ng/µL pNM10 + 20 ng/µL pNM11 + 20 ng/µL pNM12 + 2 ng/µL pMK73 + 38 ng/µL DNA ladder	100 ng/µL
**MSB903**	*unc-119(ed3) III; mirIs84 [*oxTi553] V*	*unc-119(ed3) mirIs84[rab-3p::GAL4-SK(DBD)::VP64::let-858 3'UTR+unc-122::gfp+C.br unc-119(+) *oxTi553 [eft-3p::tdTomato::H2B::unc-54 3'UTR + Cbr-unc-119(+)]]*	12.5 ng/µL crRNA (tdtomato) 12.5 ng/µL crRNA(AmpR)	existing array	
**MSB905**	*unc-119(ed3) III; mirIs85 [*oxTi700] I*	*unc-119(ed3) III; mirIs85 [nlp-12p::ChRmine + myo-2p::mCherry+ 1kb DNA ladder *oxTi700 [eft-3p::tdTomato::H2B::unc-54 3'UTR + Cbr-unc-119(+)] I*	2.8 ng/µL crRNA(tdTomato)	20 ng/µL pNM5 + 2 ng/µL pMK73 + 78 ng/µL DNA ladder	100 ng/µL
**MSB910**	*unc-119(ed3) III; mirIs86 [*oxTi553]V*	*unc-119(ed3) III; mirIs86 [*oxTi553 [eft-3p::tdTomato(truncated)::H2B::unc-54 3'UTR + Cbr-unc-119(+)]V]*	2.8 ng/µL crRNA(tdTomato)		

**MSB911**	*unc-119(ed3) III; mirIs87 [*oxTi553] V*	*unc-119(ed3) III; mirIs87[cct-2p::mtagBFP::SPE-15::spectrin::cryo2lig::wrmScarlet(1-10) + myo-2p::mCherry + 1kb DNA ladder *oxTi553 [eft-3p::tdTomato::H2B::unc-54 3'UTR + Cbr-unc-119(+)] V*	2.8 ng/µL crRNA(tdTomato)	50 ng/µL pNM10 + 1ng/µL pMK73 + 49 ng/µL DNA ladder	100 ng/µL
**MSB912**	*unc-119(ed3) III; mirIs88 [*oxTi553] V*	*unc-119(ed3) III; mirIs88[cct-2p::mtagBFP::SPE-15::spectrin::cryo2lig::wrmScarlet(1-10) + myo-2p::mCherry + 1kb DNA ladder *oxTi553 [eft-3p::tdTomato::H2B::unc-54 3'UTR + Cbr-unc-119(+)] V*	2.8 ng/µL crRNA(tdTomato)	50 ng/µL pNM11 + 1ng/µL pMK73 + 49 ng/µL DNA ladder	100 ng/µL
**MSB913**	*unc-119(ed3) III; mirIs89 [*oxTi700] I*	*unc-119(ed3) III; mirIs89[mec-4p::TRP-4::wrmScarlet(11) + myo-2p::mCherry + 1kb DNA ladder *oxTi700 [eft-3p::tdTomato::H2B::unc-54 3'UTR + Cbr-unc-119(+)]] I*	2.8 ng/µL crRNA(tdTomato)	50 ng/µL pNM11 + 1ng/µL pMK73 + 49 ng/µL DNA ladder	100 ng/µL
**MSB914**	*unc-119(ed3) III; mirIs90 [*oxTi700] I*	*unc-119(ed3) III; mirIs90[mec-4p::TRP-4::wrmScarlet(11) + myo-2p::mCherry + 1kb DNA ladder *oxTi700 [eft-3p::tdTomato::H2B::unc-54 3'UTR + Cbr-unc-119(+)]] I*	2.8 ng/µL crRNA(tdTomato)	50 ng/µL pNM11 + 1ng/µL pMK73 + 49 ng/µL DNA ladder	100 ng/µL
**MSB915**	*unc-119(ed3) III; mirIs91 [*oxTi700] I*	*unc-119(ed3) III; mirIs89[mec-4p::TRP-4::wrmScarlet(11) + myo-2p::mCherry + 1kb DNA ladder *oxTi700 [eft-3p::tdTomato::H2B::unc-54 3'UTR + Cbr-unc-119(+)]] I*	2.8 ng/µL crRNA(tdTomato)	50 ng/µL pNM11 + 1ng/µL pMK73 + 49 ng/µL DNA ladder	100 ng/µL
**MSB946**	*unc-119(ed3) III; mirIs115 [*oxTi677] II*	*unc-119(ed3) III; mirIs115[cct-2p::mtagBFP::SPE-15::spectrin::cryo2lig::wrmScarlet(1-10) + mec-4p::TRP-4::wrmScarlet(11) + myo-2p::mCherry + 1kb DNA ladder *oxTi677 [eft-3p::tdTomato::H2B::unc-54 3'UTR + Cbr-unc-119(+)]] II*	2.8 ng/µL crRNA(tdTomato)	35 ng/µL pNM10, 35 ng/µL pNM11, 2 ng/µL pMK73 + 28 ng/µL DNA ladder	100 ng/µL
**MSB952**	*unc-119(ed3) III; mirIs97 [*oxTi677] II*	*unc-119(ed3) III; mirIs97[15XUAS::ACR1 + 1kb DNA ladder *oxTi677 [eft-3p::tdTomato::H2B::unc-54 3'UTR + Cbr-unc-119(+)]] II*	2.8 ng/µL crRNA(tdTomato) 2.8 ng/µL crRNA(AmpR)	25 ng/µL pNMSB91 + 75 ng/µL DNA ladder	100 ng/µL
**MSB954**	*unc-119(ed3) III; he317; mirIs98 [*oxTi553] V*	*unc-119(ed3) III; he317[eft-3::Lox2272-BFP-Lox2272::mCherry] IV; mirIs98[ges-1p::Cre + 1kb DNA ladder *oxTi553 [eft-3p::tdTomato::H2B::unc-54 3'UTR + Cbr-unc-119(+)]] V*	2.8 ng/µL crRNA(tdTomato)	20 ng/µL pNM13 + 80 ng/µL DNA ladder	100 ng/µL
**MSB1032**	*unc-119(ed3) III; mirIs102 [*oxTi553] V, he317 IV*	*unc-119(ed3) III; mirIs102 [rab-3p::Cre + unc-122p::gfp + 1kb DNA ladder *oxTi553 [eft-3p::tdTomato::H2B::unc-54 3'UTR + Cbr-unc-119(+)]] V + he317[eft-3::Lox2272-BFP-Lox2272::mCherry] IV*	2.8 ng/µL crRNA(tdTomato)	10 ng/µL pNM14 + 50 ng/µL pCFJ68 + 30 ng/µL DNA ladder	100 ng/µL
**MSB1042**	*unc-119(ed3) III; mirIs103 [*oxTi936] X*	*unc-119(ed3) III; mirIs103[myo-3p::mCherry) + DNA ladder *oxTi936 [eft-3p::GFP::2xNLS::tbb-2 3'UTR + Cbr-unc-119(+)]] X*	crRNAs (gfp) 2.8 ng/µL each	7 ng/µL pMK23 + 93 ng/µL DNA ladder	100 ng/µL
**MSB1043**	*unc-119(ed3) III; mirIs104 [*oxTi936] X*	*unc-119(ed3) III; mirIs104[myo-3p::mCherry) + DNA ladder *oxTi936 [eft-3p::GFP::2xNLS::tbb-2 3'UTR + Cbr-unc-119(+)]] X*	crRNAs (gfp) 2.8 ng/µL each	7 ng/µL pMK23 + 93 ng/µL DNA ladder	
**MSB1047**	*unc-119(ed3) III; mirIs105 X*	*unc-119(ed3) III; mirIs105 [eft-3p::P4::2xNLS::tbb-2 3'UTR + Cbr-unc-119(+)] X]*	14 ng/µL crRNA(gfp), 3.3 ng/µL crRNA(dpy-10),	17 µM HR(gfptoP4), 9 µM HR(dpy-10)	

**MSB1087**	*unc-119(ed3) III; mirIs116 I*	*unc-119(ed3) III; mirIs116 [eft-3p::P4::2xNLS::tbb-2 3'UTR + Cbr-unc-119(+)] I]*	14 ng/µL crRNA(gfp), 3.3 ng/µL crRNA(dpy-10),	17 µM HR(gfptoP4), 9 µM HR(dpy-10)	
**MSB1088**	*unc-119(ed3) III; mirIs110 [*oxTi553] V*	*mirIs110 [odr-7p::NL250+unc-122p::GFP *oxTi553 [eft-3p::tdTomato::H2B::unc-54 3'UTR + Cbr-unc-119(+)]] V*	12.5 ng/µL crRNA(tdTomato) 12.5 ng/µL crRNA(AmpR)	100 ng/µL pNMSB130, 20 ng/µL pCFJ68	120 ng/µL
**MSB1089**	*unc-119(ed3) III; mirIs111 [*oxTi553] V*	*mirIs111 [odr-7p::NL250+unc-122p::GFP *oxTi553 [eft-3p::tdTomato::H2B::unc-54 3'UTR + Cbr-unc-119(+)]] V*	12.5 ng/µL crRNA(tdTomato) 12.5 ng/µL crRNA(AmpR)	100 ng/µL pNMSB130, 20 ng/µL pCFJ68	120 ng/µL
**MSB1090**	*unc-119(ed3) III; mirIs112 [*oxTi553] V*	*mirIs112 [odr-7p::NL250+unc-122p::GFP *oxTi553 [eft-3p::tdTomato::H2B::unc-54 3'UTR + Cbr-unc-119(+)]] V*	12.5 ng/µL crRNA(tdTomato) 12.5 ng/µL crRNA(AmpR)	100 ng/µL pNMSB130, 20 ng/µL pCFJ68	120 ng/µL
**MSB1104**	*unc-119(ed3) III; mirIs117 [*oxTi553] V*	*unc-119(ed3) III; mirIs117 [myo-2p::mCherry + DNA ladder *oxTi553 [eft-3p::tdTomato::H2B::unc-54 3'UTR + Cbr-unc-119(+)]] V*	2.8 ng/µL crRNA(tdTomato)	2 ng/µL pMK73 + 98 ng/µL empty vector	100 ng/µL
**MSB1105**	*unc-119(ed3) III; mirIs118 [*oxTi553] V*	*unc-119(ed3) III; mirIs118 [myo-2p::mCherry + DNA ladder *oxTi553 [eft-3p::tdTomato::H2B::unc-54 3'UTR + Cbr-unc-119(+)]] V*	2.8 ng/µL crRNA(tdTomato)	2 ng/µL pMK73 + 98 ng/µL empty vector	100 ng/µL
**MSB1106**	*unc-119(ed3) III; mirIs119 [*oxTi553] V*	*unc-119(ed3) III; mirIs119 [myo-2p::mCherry + DNA ladder *oxTi553 [eft-3p::tdTomato::H2B::unc-54 3'UTR + Cbr-unc-119(+)]] V*	2.8 ng/µL crRNA(tdTomato)	2 ng/µL pMK73 + 98 ng/µL empty vector	100 ng/µL
**MSB1107**	*unc-119(ed3) III; mirIs120 [*oxTi553] V*	*unc-119(ed3) III; mirIs120 [myo-2p::mCherry + DNA ladder *oxTi553 [eft-3p::tdTomato::H2B::unc-54 3'UTR + Cbr-unc-119(+)]] V*	2.8 ng/µL crRNA(tdTomato)	2 ng/µL pMK73 + 98 ng/µL empty vector	100 ng/µL
**MSB1108**	*unc-119(ed3) III; mirIs121 [*oxTi553] V*	*unc-119(ed3) III; mirIs121 [myo-2p::mCherry + DNA ladder *oxTi553 [eft-3p::tdTomato::H2B::unc-54 3'UTR + Cbr-unc-119(+)]] V*	2.8 ng/µL crRNA(tdTomato)	2 ng/µL pMK73 + 98 ng/µL empty vector	100 ng/µL
**MSB1109**	*unc-119(ed3) III; mirIs122 [*oxTi553] V*	*unc-119(ed3) III; mirIs122 [myo-2p::mCherry + DNA ladder *oxTi553 [eft-3p::tdTomato::H2B::unc-54 3'UTR + Cbr-unc-119(+)]] V*	2.8 ng/µL crRNA(tdTomato)	2 ng/µL pMK73 + 98 ng/µL empty vector	100 ng/µL
**MSB1110**	*unc-119(ed3) III; mirIs123 [*oxTi553] V*	*unc-119(ed3) III; mirIs123 [myo-2p::mCherry + DNA ladder *oxTi553 [eft-3p::tdTomato::H2B::unc-54 3'UTR + Cbr-unc-119(+)]] V*	2.8 ng/µL crRNA(tdTomato)	2 ng/µL pMK73 + 98 ng/µL empty vector	100 ng/µL
**MSB1111**	*unc-119(ed3) III; mirIs124 [*oxTi553] V*	*unc-119(ed3) III; mirIs124 [myo-2p::mCherry + DNA ladder *oxTi553 [eft-3p::tdTomato::H2B::unc-54 3'UTR + Cbr-unc-119(+)]] V*	2.8 ng/µL crRNA(tdTomato)	2 ng/µL pMK73 + 98 ng/µL empty vector	100 ng/µL
**MSB1112**	*unc-119(ed3) III; mirIs125 [*oxTi553] V*	*unc-119(ed3) III; mirIs125 [myo-2p::mCherry + DNA ladder *oxTi553 [eft-3p::tdTomato::H2B::unc-54 3'UTR + Cbr-unc-119(+)]] V*	2.8 ng/µL crRNA(tdTomato)	2 ng/µL pMK73 + 98 ng/µL empty vector	100 ng/µL

**MSB1113**	*unc-119(ed3) III; mirIs126 [*oxTi553] V*	*unc-119(ed3) III; mirIs126 [myo-2p::mCherry + DNA ladder *oxTi553 [eft-3p::tdTomato::H2B::unc-54 3'UTR + Cbr-unc-119(+)]] V*	2.8 ng/µL crRNA(tdTomato)	2 ng/µL pMK73 + 98 ng/µL empty vector	100 ng/µL
**MSB1115**	*unc-119(ed3) III; mirIs127 [*oxTi677] II*	*unc-119(ed3) III; mirIs127[myo-2p::mCherry + DNA ladder *oxTi677 [eft-3p::tdTomato::H2B::unc-54 3'UTR + Cbr-unc-119(+)]] II*	2.8 ng/µL crRNA(tdTomato)	2 ng/µL pMK73 + 98 ng/µL empty vector	100 ng/µL
**MSB1116**	*unc-119(ed3) III; mirIs128 [*oxTi677] II*	*unc-119(ed3) III; mirIs128[myo-2p::mCherry + DNA ladder*oxTi677 [eft-3p::tdTomato::H2B::unc-54 3'UTR + Cbr-unc-119(+)]] II*	2.8 ng/µL crRNA(tdTomato)	2 ng/µL pMK73 + 98 ng/µL empty vector	100 ng/µL
**MSB1117**	*unc-119(ed3) III; mirIs129 [*oxTi677] II*	*unc-119(ed3) III; mirIs129[myo-2p::mCherry + DNA ladder *oxTi677 [eft-3p::tdTomato::H2B::unc-54 3'UTR + Cbr-unc-119(+)]] II*	2.8 ng/µL crRNA(tdTomato)	2 ng/µL pMK73 + 98 ng/µL empty vector	100 ng/µL
**MSB1118**	*unc-119(ed3) III; mirIs130 [*oxTi546] III*	*unc-119(ed3) III; mirIs130[myo-2p::mCherry + DNA ladder *oxTi546 [eft-3p::tdTomato::H2B::unc-54 3'UTR + Cbr-unc-119(+)]] III*	2.8 ng/µL crRNA(tdTomato)	2 ng/µL pMK73 + 98 ng/µL empty vector	100 ng/µL
**MSB1119**	*unc-119(ed3) III; mirIs131 [*oxTi546] III*	*unc-119(ed3) III; mirIs131[myo-2p::mCherry + DNA ladder *oxTi546 [eft-3p::tdTomato::H2B::unc-54 3'UTR + Cbr-unc-119(+)]] III*	2.8 ng/µL crRNA(tdTomato)	2 ng/µL pMK73 + 98 ng/µL empty vector	100 ng/µL
**MSB1120**	*unc-119(ed3) III; mirIs132 [*oxTi546] III*	*unc-119(ed3) III; mirIs132[myo-2p::mCherry + DNA ladder *oxTi546 [eft-3p::tdTomato::H2B::unc-54 3'UTR + Cbr-unc-119(+)]] III*	2.8 ng/µL crRNA(tdTomato)	2 ng/µL pMK73 + 98 ng/µL empty vector	100 ng/µL
**MSB1121**	*unc-119(ed3) III; mirIs133 [*oxTi546] III*	*unc-119(ed3) III; mirIs133[myo-2p::mCherry + DNA ladder *oxTi546 [eft-3p::tdTomato::H2B::unc-54 3'UTR + Cbr-unc-119(+)]] III*	2.8 ng/µL crRNA(tdTomato)	2 ng/µL pMK73 + 98 ng/µL empty vector	100 ng/µL
**MSB1122**	*unc-119(ed3) III; mirIs134 [*oxTi553] V*	*unc-119(ed3) III; mirIs134[myo-2p::mCherry + DNA ladder *oxTi553 [eft-3p::tdTomato::H2B::unc-54 3'UTR + Cbr-unc-119(+)]] V*	2.8 ng/µL crRNA(tdTomato)	2 ng/µL pMK73 + 98 ng/µL empty vector	100 ng/µL
**MSB1123**	*unc-119(ed3) III; mirIs135 [*oxTi553] V*	*unc-119(ed3) III; mirIs135[myo-2p::mCherry + DNA ladder *oxTi553 [eft-3p::tdTomato::H2B::unc-54 3'UTR + Cbr-unc-119(+)]] V*	2.8 ng/µL crRNA(tdTomato)	2 ng/µL pMK73 + 98 ng/µL empty vector	100 ng/µL
**MSB1124**	*unc-119(ed3) III; mirIs136 [*oxTi553] V*	*unc-119(ed3) III; mirIs136[myo-2p::mCherry + DNA ladder *oxTi553 [eft-3p::tdTomato::H2B::unc-54 3'UTR + Cbr-unc-119(+)]] V*	2.8 ng/µL crRNA(tdTomato)	2 ng/µL pMK73 + 98 ng/µL empty vector	100 ng/µL
**MSB1125**	*unc-119(ed3) III; mirIs137 [*oxTi553] V*	*unc-119(ed3) III; mirIs137[myo-2p::mCherry + DNA ladder *oxTi553 [eft-3p::tdTomato::H2B::unc-54 3'UTR + Cbr-unc-119(+)]] V*	2.8 ng/µL crRNA(tdTomato)	2 ng/µL pMK73 + 98 ng/µL empty vector	100 ng/µL
**MSB1164**	*unc-119(ed3) III; mirIs139 [*oxTi553] V*	*unc-119(ed3) III; mirIs139[unc-122p::GFP + empty vector *oxTi553 [eft-3p::tdTomato::H2B::unc-54 3'UTR + Cbr-unc-119(+)]] V*	1.4 ng/µL crRNA(tdTomato)	50 ng/µL pCFJ68 + 170 ng/µL empty vector	100 ng/µL
**MSB1165**	*unc-119(ed3) III; mirIs140 [*oxTi553] V*	*unc-119(ed3) III; mirIs140[unc-122p::GFP + empty vector *oxTi553 [eft-3p::tdTomato::H2B::unc-54 3'UTR + Cbr-unc-119(+)]] V*	1.4 ng/µL crRNA(tdTomato)	50 ng/µL pCFJ68 + 170 ng/µL empty vector	220 ng/µL

MSB1177	*unc-119(ed3) III; mirIs142[*oxTi556] I*	*unc-119(ed3) III; mirIs142[myo-2p::mCherry + empty vector *oxTi556 [eft-3p::tdTomato::H2B::unc-54 3'UTR + Cbr-unc-119(+)]] I*	1.4 ng/µL crRNA(tdTomato)	2 ng/µL pMK73 + 98 ng/µL empty vector	100 ng/µL
MSB1178	*unc-119(ed3) III; mirIs143 [*oxTi556] I*	*unc-119(ed3) III; mirIs143[myo-2p::mCherry + empty vector *oxTi556 [eft-3p::tdTomato::H2B::unc-54 3'UTR + Cbr-unc-119(+)]] I*	1.4 ng/µL crRNA(tdTomato)	2 ng/µL pMK73 + 98 ng/µL empty vector	100 ng/µL
**MSB1179**	*unc-119(ed3) III; mirIs144 [*oxTi556] I*	*unc-119(ed3) III; mirIs144[myo-2p::mCherry + empty vector *oxTi556 [eft-3p::tdTomato::H2B::unc-54 3'UTR + Cbr-unc-119(+)]] I*	1.4 ng/µL crRNA(tdTomato)	2 ng/µL pMK73 + 98 ng/µL empty vector	100 ng/µL
**MSB1180**	*unc-119(ed3) III; mirIs145 [*oxTi556] I*	*unc-119(ed3) III; mirIs145[myo-2p::mCherry + empty vector *oxTi556 [eft-3p::tdTomato::H2B::unc-54 3'UTR + Cbr-unc-119(+)]] I*	1.4 ng/µL crRNA(tdTomato)	2 ng/µL pMK73 + 98 ng/µL empty vector	100 ng/µL
**MSB1181**	*unc-119(ed3) III; mirIs146 [*oxTi556] I*	*unc-119(ed3) III; mirIs146[myo-2p::mCherry + empty vector *oxTi556 [eft-3p::tdTomato::H2B::unc-54 3'UTR + Cbr-unc-119(+)]] I*	1.4 ng/µL crRNA(tdTomato)	2 ng/µL pMK73 + 98 ng/µL empty vector	100 ng/µL
**MSB1182**	*unc-119(ed3) III; mirIs147 [*oxTi564] II*	*unc-119(ed3) III; mirIs147[myo-2p::mCherry + empty vector *oxTi564 [eft-3p::tdTomato::H2B::unc-54 3'UTR + Cbr-unc-119(+)]] II*	1.4 ng/µL crRNA(tdTomato)	2 ng/µL pMK73 + 98 ng/µL empty vector	100 ng/µL
**MSB1183**	*unc-119(ed3) III; mirIs148 [*oxTi564] II*	*unc-119(ed3) III; mirIs148[myo-2p::mCherry + empty vector *oxTi564 [eft-3p::tdTomato::H2B::unc-54 3'UTR + Cbr-unc-119(+)]] II*	1.4 ng/µL crRNA(tdTomato)	2 ng/µL pMK73 + 98 ng/µL empty vector	100 ng/µL
**MSB1184**	*unc-119(ed3) III; mirIs149 [*oxTi564] II*	*unc-119(ed3) III; mirIs149[myo-2p::mCherry + empty vector *oxTi564 [eft-3p::tdTomato::H2B::unc-54 3'UTR + Cbr-unc-119(+)]] II*	1.4 ng/µL crRNA(tdTomato)	2 ng/µL pMK73 + 98 ng/µL empty vector	100 ng/µL
**MSB1185**	*unc-119(ed3) III; mirIs150 [*oxTi564] II*	*unc-119(ed3) III; mirIs150[myo-2p::mCherry + empty vector *oxTi564 [eft-3p::tdTomato::H2B::unc-54 3'UTR + Cbr-unc-119(+)]] II*	1.4 ng/µL crRNA(tdTomato)	2 ng/µL pMK73 + 98 ng/µL empty vector	100 ng/µL
**MSB1186**	*unc-119(ed3) III; mirIs151 [*oxTi564] II*	*unc-119(ed3) III; mirIs151[myo-2p::mCherry + empty vector *oxTi564 [eft-3p::tdTomato::H2B::unc-54 3'UTR + Cbr-unc-119(+)]] II*	1.4 ng/µL crRNA(tdTomato)	2 ng/µL pMK73 + 98 ng/µL empty vector	100 ng/µL
**MSB1187**	*unc-119(ed3) III; mirIs152 [*oxTi564] II*	*unc-119(ed3) III; mirIs152[myo-2p::mCherry + empty vector *oxTi564 [eft-3p::tdTomato::H2B::unc-54 3'UTR + Cbr-unc-119(+)]] II*	1.4 ng/µL crRNA(tdTomato)	2 ng/µL pMK73 + 98 ng/µL empty vector	100 ng/µL
**MSB1188**	*unc-119(ed3) III; mirIs153 [*oxTi619] III*	*unc-119(ed3) III; mirIs153[myo-2p::mCherry + empty vector *oxTi619 [eft-3p::tdTomato::H2B::unc-54 3'UTR + Cbr-unc-119(+)]] III*	1.4 ng/µL crRNA(tdTomato)	2 ng/µL pMK73 + 98 ng/µL empty vector	100 ng/µL
**MSB1189**	*unc-119(ed3) III; mirIs154 [*oxTi619] III*	*unc-119(ed3) III; mirIs154[myo-2p::mCherry + empty vector *oxTi619 [eft-3p::tdTomato::H2B::unc-54 3'UTR + Cbr-unc-119(+)]] III*	1.4 ng/µL crRNA(tdTomato)	2 ng/µL pMK73 + 98 ng/µL empty vector	100 ng/µL
**MSB1190**	*unc-119(ed3) III; mirIs155 [*oxTi619] III*	*unc-119(ed3) III; mirIs155[myo-2p::mCherry + empty vector *oxTi619 [eft-3p::tdTomato::H2B::unc-54 3'UTR + Cbr-unc-119(+)]] III*	1.4 ng/µL crRNA(tdTomato)	2 ng/µL pMK73 + 98 ng/µL empty vector	100 ng/µL

**MSB1191**	*unc-119(ed3) III; mirIs156 [*oxTi619] III*	*unc-119(ed3) III; mirIs156[myo-2p::mCherry + empty vector *oxTi619 [eft-3p::tdTomato::H2B::unc-54 3'UTR + Cbr-unc-119(+)]] III*	1.4 ng/µL crRNA(tdTomato)	2 ng/µL pMK73 + 98 ng/µL empty vector	100 ng/µL
**MSB1192**	*unc-119(ed3) III; mirIs157 [*oxTi619] III*	*unc-119(ed3) III; mirIs157[myo-2p::mCherry + empty vector *oxTi619 [eft-3p::tdTomato::H2B::unc-54 3'UTR + Cbr-unc-119(+)]] III*	1.4 ng/µL crRNA(tdTomato)	2 ng/µL pMK73 + 98 ng/µL empty vector	100 ng/µL
**MSB1193**	*unc-119(ed3) III; mirIs158 [*oxTi619] III*	*unc-119(ed3) III; mirIs158[myo-2p::mCherry + empty vector *oxTi619 [eft-3p::tdTomato::H2B::unc-54 3'UTR + Cbr-unc-119(+)]] III*	1.4 ng/µL crRNA(tdTomato)	2 ng/µL pMK73 + 98 ng/µL empty vector	100 ng/µL
**MSB1194**	*unc-119(ed3) III; mirIs159 [*oxTi619] III*	*unc-119(ed3) III; mirIs159[myo-2p::mCherry + empty vector *oxTi619 [eft-3p::tdTomato::H2B::unc-54 3'UTR + Cbr-unc-119(+)]] III*	1.4 ng/µL crRNA(tdTomato)	2 ng/µL pMK73 + 98 ng/µL empty vector	100 ng/µL
**MSB1195**	*unc-119(ed3) III; mirIs160 [*oxTi619] III*	*unc-119(ed3) III; mirIs160[myo-2p::mCherry + empty vector *oxTi619 [eft-3p::tdTomato::H2B::unc-54 3'UTR + Cbr-unc-119(+)]] III*	1.4 ng/µL crRNA(tdTomato)	2 ng/µL pMK73 + 98 ng/µL empty vector	100 ng/µL
**MSB1196**	*unc-119(ed3) III; mirIs161 [*oxTi619] III*	*unc-119(ed3) III; mirIs161[myo-2p::mCherry + empty vector *oxTi619 [eft-3p::tdTomato::H2B::unc-54 3'UTR + Cbr-unc-119(+)]] III*	1.4 ng/µL crRNA(tdTomato)	2 ng/µL pMK73 + 98 ng/µL empty vector	100 ng/µL
**MSB1197**	*unc-119(ed3) III; mirIs162 [*oxTi619] III*	*unc-119(ed3) III; mirIs162[myo-2p::mCherry + empty vector *oxTi619 [eft-3p::tdTomato::H2B::unc-54 3'UTR + Cbr-unc-119(+)]] III*	1.4 ng/µL crRNA(tdTomato)	2 ng/µL pMK73 + 98 ng/µL empty vector	100 ng/µL
**MSB1198**	*unc-119(ed3) III; mirIs163 [*oxTi705] IV*	*unc-119(ed3) III; mirIs163[myo-2p::mCherry + empty vector *oxTi705 [eft-3p::tdTomato::H2B::unc-54 3'UTR + Cbr-unc-119(+)]] IV*	1.4 ng/µL crRNA(tdTomato)	2 ng/µL pMK73 + 98 ng/µL empty vector	100 ng/µL
**MSB1199**	*unc-119(ed3) III; mirIs164[*oxTi705] IV*	*unc-119(ed3) III; mirIs164[myo-2p::mCherry + empty vector *oxTi705 [eft-3p::tdTomato::H2B::unc-54 3'UTR + Cbr-unc-119(+)]] IV*	1.4 ng/µL crRNA(tdTomato)	2 ng/µL pMK73 + 98 ng/µL empty vector	100 ng/µL
**MSB1200**	*unc-119(ed3) III; mirIs165 [*oxTi543] V*	*unc-119(ed3) III; mirIs165[myo-2p::mCherry + empty vector *oxTi543 [eft-3p::tdTomato::H2B::unc-54 3'UTR + Cbr-unc-119(+)]] V*	1.4 ng/µL crRNA(tdTomato)	2 ng/µL pMK73 + 98 ng/µL empty vector	100 ng/µL
**MSB1201**	*unc-119(ed3) III; mirIs166 [*oxTi543] V*	*unc-119(ed3) III; mirIs166[myo-2p::mCherry + empty vector *oxTi543 [eft-3p::tdTomato::H2B::unc-54 3'UTR + Cbr-unc-119(+)]] V*	1.4 ng/µL crRNA(tdTomato)	2 ng/µL pMK73 + 98 ng/µL empty vector	100 ng/µL
**MSB1202**	*unc-119(ed3) III; mirIs167 [*oxTi668] X*	*unc-119(ed3) III; mirIs167[myo-2p::mCherry + empty vector *oxTi668 [eft-3p::tdTomato::H2B::unc-54 3'UTR + Cbr-unc-119(+)]] X*	1.4 ng/µL crRNA(tdTomato)	2 ng/µL pMK73 + 98 ng/µL empty vector	100 ng/µL
**MSB1203**	*unc-119(ed3) III; mirI168 [*oxTi668] X*	*unc-119(ed3) III; mirIs168[myo-2p::mCherry + empty vector *oxTi668 [eft-3p::tdTomato::H2B::unc-54 3'UTR + Cbr-unc-119(+)]] X*	1.4 ng/µL crRNA(tdTomato)	2 ng/µL pMK73 + 98 ng/µL empty vector	100 ng/µL
**MSB1204**	*unc-119(ed3) III; mirIs169 [*oxTi668] X*	*unc-119(ed3) III; mirIs169[myo-2p::mCherry + empty vector *oxTi668 [eft-3p::tdTomato::H2B::unc-54 3'UTR + Cbr-unc-119(+)]] X*	1.4 ng/µL crRNA(tdTomato)	2 ng/µL pMK73 + 98 ng/µL empty vector	100 ng/µL

**MSB1205**	*unc-119(ed3) III; mirIs170 [*oxTi668] X*	*unc-119(ed3) III; mirIs170[myo-2p::mCherry + empty vector *oxTi668 [eft-3p::tdTomato::H2B::unc-54 3'UTR + Cbr-unc-119(+)]] X*	1.4 ng/µL crRNA(tdTomato)	2 ng/µL pMK73 + 98 ng/µL empty vector	100 ng/µL
**MSB1206**	*unc-119(ed3) III; mirIs171 [*oxTi668] X*	*unc-119(ed3) III; mirIs171[myo-2p::mCherry + empty vector *oxTi668 [eft-3p::tdTomato::H2B::unc-54 3'UTR + Cbr-unc-119(+)]] X*	1.4 ng/µL crRNA(tdTomato)	2 ng/µL pMK73 + 98 ng/µL empty vector	100 ng/µL
**MSB1207**	*unc-119(ed3) III; mirIs172 [*oxTi668] X*	*unc-119(ed3) III; mirIs172[myo-2p::mCherry + empty vector *oxTi668 [eft-3p::tdTomato::H2B::unc-54 3'UTR + Cbr-unc-119(+)]] X*	1.4 ng/µL crRNA(tdTomato)	2 ng/µL pMK73 + 98 ng/µL empty vector	100 ng/µL
**MSB1208**	*unc-119(ed3) III; mirIs173 [*oxTi668] X*	*unc-119(ed3) III; mirIs173[myo-2p::mCherry + empty vector *oxTi668 [eft-3p::tdTomato::H2B::unc-54 3'UTR + Cbr-unc-119(+)]] X*	1.4 ng/µL crRNA(tdTomato)	2 ng/µL pMK73 + 98 ng/µL empty vector	100 ng/µL
**MSB1209**	*unc-119(ed3) III; mirIs174 [*oxTi668] X*	*unc-119(ed3) III; mirIs174[myo-2p::mCherry + empty vector *oxTi668 [eft-3p::tdTomato::H2B::unc-54 3'UTR + Cbr-unc-119(+)]] X*	1.4 ng/µL crRNA(tdTomato)	2 ng/µL pMK73 + 98 ng/µL empty vector	100 ng/µL
**MSB1210**	*unc-119(ed3) III; mirIs175 [*oxTi668] X*	*unc-119(ed3) III; mirIsxxx[myo-2p::mCherry + empty vector *oxTi668 [eft-3p::tdTomato::H2B::unc-54 3'UTR + Cbr-unc-119(+)]] X*	1.4 ng/µL crRNA(tdTomato)	2 ng/µL pMK73 + 98 ng/µL empty vector	100 ng/µL
**MSB1211**	*unc-119(ed3) III; mirIs176 [*oxTi668] X*	*unc-119(ed3) III; mirIsxxx[myo-2p::mCherry + empty vector *oxTi668 [eft-3p::tdTomato::H2B::unc-54 3'UTR + Cbr-unc-119(+)]] X*	1.4 ng/µL crRNA(tdTomato)	2 ng/µL pMK73 + 98 ng/µL empty vector	100 ng/µL
**MSB1212**	*unc-119(ed3) III; mirIs177 [*oxTi700] I*	*unc-119(ed3) III; mirIs177[myo-2p::mCherry + empty vector *oxTi700 [eft-3p::tdTomato::H2B::unc-54 3'UTR + Cbr-unc-119(+)]] I*	1.4 ng/µL crRNA(tdTomato)	2 ng/µL pMK73 + 98 ng/µL empty vector	100 ng/µL
**MSB1213**	*unc-119(ed3) III; mirIs178 [*oxTi668] I*	*unc-119(ed3) III; mirIs178[myo-2p::mCherry + empty vector *oxTi700 [eft-3p::tdTomato::H2B::unc-54 3'UTR + Cbr-unc-119(+)]] I*	1.4 ng/µL crRNA(tdTomato)	2 ng/µL pMK73 + 98 ng/µL empty vector	100 ng/µL
**MSB1214**	*unc-119(ed3) III; mirIs179 [*oxTi677] II*	*unc-119(ed3) III; mirIs179[myo-2p::mCherry + empty vector *oxTi677 [eft-3p::tdTomato::H2B::unc-54 3'UTR + Cbr-unc-119(+)]] II*	1.4 ng/µL crRNA(tdTomato)	2 ng/µL pMK73 + 98 ng/µL empty vector	100 ng/µL
**MSB1215**	*unc-119(ed3) III; mirIs180 [*oxTi546] III*	*unc-119(ed3) III; mirIs180[myo-2p::mCherry + empty vector *oxTi546 [eft-3p::tdTomato::H2B::unc-54 3'UTR + Cbr-unc-119(+)]] III*	1.4 ng/µL crRNA(tdTomato)	2 ng/µL pMK73 + 98 ng/µL empty vector	100 ng/µL
**MSB1216**	*unc-119(ed3) III; mirIs181 [*oxTi546] III*	*unc-119(ed3) III; mirIs181[myo-2p::mCherry + empty vector *oxTi546 [eft-3p::tdTomato::H2B::unc-54 3'UTR + Cbr-unc-119(+)]] III*	1.4 ng/µL crRNA(tdTomato)	2 ng/µL pMK73 + 98 ng/µL empty vector	100 ng/µL
