# Supplementary material for: FLInt: single shot safe harbor transgene integration via Fluorescent Landmark Interference
Source: G3 (Bethesda). 2023 Feb 20;13(5):jkad041. doi: 10.1093/g3journal/jkad041 (PMC10151404; doi:10.1093/g3journal/jkad041)
Supplement: jkad041_Supplementary_Data [file jkad041_supplementary_data.zip › Supplemental_Table_S3-7_G3-2022-404006.pdf]

Supplemental Tables S3-S7

Table S3: Plasmid(s)

pCFJ68 (*unc-122*p::*gfp*)	From AddGene
pMK23 (*myo-3*p::*mCherry*)	From NMSB lab
pMK73 (*myo-2*p::*mCherry*)	From NMSB lab
pNM5 (*nlp-12*p::*ChRmine*)	From NMSB lab
pNM10(*cct-2*p::*mtagBFP2*::*spe-15*::*spectrin*::*cryolig2*:: *wrmScarlet1-10*)	From NMSB lab
pNM11(*mec-4*p::*trp-4*::*wrmScarlet 11*)	From NMSB lab
pNM12 (*mec-4*p::*RGECO1*)	From NMSB lab
pNM13 (*ges-1*p::*CRE*)	From NMSB lab
pNM14 (rab-3p::CRE)	From NMSB lab
pNMSB91 (*15xUAS::delta pes-10p::ACR1*)	From NMSB lab
pHW393 (*rab-3p::gal4*)	From NMSB lab

Table S4: DNA primer(s) (purchased from IDT)

Primer	Sequence (5´to 3´)
tdTomato genotyping FWD1	TTACTTGTACAGCTCGTCCATGC
tdTomato genotyping FWD2	GACCCAGGACTCCTCCCT
tdTomato genotyping REV	TTTATAATGAGGTCAAACATTCAGTCCCAGCGTTTT
lat-1::loxP genotyping FWD	CGATGTTGACAACTGAAGTGA
lat-1::loxP genotyping REV	GGTAATTTCTGACATGGCTCA
cry2olig genotyping FWD	TTGATCGCTTGGACAATCCCGCGTTAC
cry2olig genotyping REV	GAGATGCTAGTACTCGTTGTGGGAGG
C55B7.3 genotyping FWD	AAAGACGTTGGATGCCTT
C55B7.3 genotyping REV	CACAATACACAGACAGCAACT
mCherry amplification FWD	AGAAGGCCGACCATATGAGGGAACACAA
mCherry amplification REV	GCGTCACTGTAACAACTCCTCCGTCTTCAA
rps-15 amplification FWD	TCACACCATCCGTCGTCTCTG
rps-15 amplification REV	GCGTCACTGTAACAACTCCTCCGTCTTCAA

Red primers were used for qPCR.

Table S5: crRNA(s) (purchased from IDT)

**crRNA**	**Sequence (5´to 3´)**
crRNA (*tdTomato*)	GTGATGAACTTCGAGGACGG
crRNA (*gfp1*)	TGAACTATACAAATGCCCGG
crRNA (*gfp2*)	CTTGTCACTACTTTCTGTTA
crRNA (*AmpR*)	ATTAATAGACTGGATGGAGG

Table S6: HR template(s) (purchased from IDT)

**HR template**	**Sequence (5´to 3´)**
HR template (*gfp-to-P4*)	TTAAATTTTCAGCCAACACTTGTCACTACTTTCTGTCATGGTGTT CAATGCTTCTCGAGATACCCAGATCATAT
HR template (*lat-1*::*loxP*)	TGAACAAGCCGAGCATGTATTGTCAAGATTTATAGATAACTTCG TATAGCATACATTATACGAAGTTATATGGGCCCTAAAAAGAAGC GTAAAGCTTCCAAGGGAGAGGAGGACAACATGGCCATCATCAA GGAGTTCATGCGTTTCAAGGCCGAGGGACGTCTGCATATCTTTG ATGCGTGTACATTCTCCCTTTTTCT

Table S7*: tdTomato* CRISPR mix

**Reagents**	**Initial conc.**	**Desired con.**	**Vol. used**
crRNA(*tdTomato*)	167 µM	14 µM	1 µL
tracrRNA	167 µM	14 µM	1 µL
milliQ water	-		7 µL
Incubate at 95°C for 5 min			
Incubate at RT for 5 min			
Add 1µL Cas9			
Incubate at RT for 5 min			
Aliquot into PCR tubes (2 µL each)			
Store at -20°C for further use			
